# Automatic Real-Time Pose Estimation of Machinery from Images

**DOI:** 10.3390/s22072627

**Published:** 2022-03-29

**Authors:** Marcel Bertels, Boris Jutzi, Markus Ulrich

**Affiliations:** Institute of Photogrammetry and Remote Sensing (IPF), Karlsruhe Institute of Technology, 76128 Karlsruhe, Germany; marcel.bertels@alumni.kit.edu (M.B.); boris.jutzi@kit.edu (B.J.)

**Keywords:** machine vision, stereo camera system, localization, real-time, pose estimation, marker detection

## Abstract

The automatic positioning of machines in a large number of application areas is an important aspect of automation. Today, this is often done using classic geodetic sensors such as Global Navigation Satellite Systems (GNSS) and robotic total stations. In this work, a stereo camera system was developed that localizes a machine at high frequency and serves as an alternative to the previously mentioned sensors. For this purpose, algorithms were developed that detect active markers on the machine in a stereo image pair, find stereo point correspondences, and estimate the pose of the machine from these. Theoretical influences and accuracies for different systems were estimated with a Monte Carlo simulation, on the basis of which the stereo camera system was designed. Field measurements were used to evaluate the actual achievable accuracies and the robustness of the prototype system. The comparison is present with reference measurements with a laser tracker. The estimated object pose achieved accuracies higher than 16 mm with the translation components and accuracies higher than 3 mrad with the rotation components. As a result, 3D point accuracies higher than 16 mm were achieved by the machine. For the first time, a prototype could be developed that represents an alternative, powerful image-based localization method for machines to the classical geodetic sensors.

## 1. Introduction

Firstly, the motivation for the development of an image-based prototype for the localization of machines is presented. Subsequently, the state of the art is described in more detail.

### 1.1. Motivation

Today, the positioning of construction machinery on construction sites is mainly done automatically via robotic total stations by precisely measuring angle and distance [1,2]. Unfortunately, precise robotic total stations are very expensive: they are several tens of thousands of USD. In contrast, camera sensors cost only a fraction of robotic total stations due to their distribution in the market worldwide. Therefore, more and more camera systems are being used in industrial and geodetic applications. Another major advantage of cameras over robotic total stations is the frequency at which the sensors can measure. While the frequency of robotic total stations typically is 10 Hz and can be increased up to 20 Hz [3], camera frame rates of 500 Hz are not uncommon nowadays [4]. It is important to consider not only the purely technically possible measuring frequency, but also the feasible tracking speed. This is arbitrarily large in the field of view (FOV) of a camera system. In contrast, robotic total stations are limited by their turning speed [5]. This turning speed is up to 180 deg/s [6,7].

In this work, a prototype system based on cameras was developed that can automatically, continuously, and accurately locate a wide variety of machines. This includes construction machines such as excavators, caterpillars, and graders, but also cargo loaders for aircraft, for example. For this purpose, 10–50 m is defined as the operating range of the system in which at should be able to estimate an object’s pose, i.e., the object’s position and orientation in 3D space. The system should therefore, be as generic as possible to stay versatile. Therefore, we also designed the trials to be as generic as possible within the application scope. For example, we did not use a special type of machine for the experiments, but performed the experiments with a steel frame which served as a generic prototype. This allows the results to be applicable to as many different types of machines as possible. The prototype is also to be modified in such a way that it will operate reliably under adverse environmental conditions and even in the dark. The accuracy of such a system for construction machinery should be in the low centimeter range for the 3D position, as defined in [1]. In addition, the system should operate in real time, which in the context of construction machinery is assumed to be more than 1 Hz.

The use of RGBD or ToF cameras is not suitable for the outlined application. Modern RGBD cameras such as Intel RealSense or Microsoft Kinect have only small working ranges and are designed for close-range applications. Distances of up to 50 m are not feasible with these systems [8,9]. Intel RealSense, for example, has a recommended working range of up to 6 m [10]. Current ToF cameras are also only designed for close-range work and have a working ranges of less than 10 m [11,12]. There are also commercial stereo cameras available that provide long-range measurements of objects up to 70 m away in outdoor environments, such as SceneScanPro (Nerian Vision Technologies) [13], ZED2 (Stereolabs) [14], and Omega (Arcure) [15]. They compute a dense distance map, which is not relevant for our application, as we will show later. Furthermore, the low resolution of up to four megapixels and distance values that are only provided for integer pixel positions result in accuracies that are far from sufficient for our application. Additionally, they would not work well in low-light conditions, which is a necessary requirement to cover a wide variety of applications.

The prototype performs the determination of an object’s pose using a camera system. In contrast, the integration of the camera system into the higher-level reference system is not the subject of the work. In order for the camera system to be able to serve as a replacement for current systems that use robotic total stations, it is important that the requirements regarding accuracy, robustness, and applicability are the same for the camera system as for the current systems. It is mandatory for the camera system to operate automatically and calculate the pose of the machine without user interaction.

### 1.2. State of the Art

In modern construction machine control with *robotic total stations*, these are used in combination with 2-axis inclination sensors to estimate the poses of the machines. The disadvantage of this is the synchronization of the different sensors for the estimation of the pose, and low target distances of up to 50 m by the robotic total stations. As an alternative, Global Navigation Satellite System (GNSS) sensors are often used for pose estimation. However, these usually have insufficient accuracy in the height component, which is why they cannot be used in many cases [1]. Moreover, their application requires robust satellite reception, which often cannot be guaranteed [1].

For the determination of 3D coordinates of discrete points, *circular markers* are preferably used in many applications [4]. These allow a high contrast in a small space and the center of the circle can be determined very precisely in the image. A distinction must be made between passive and active markers. Passive markers can be made of retro-reflective or diffusely reflective materials and can be illuminated by LED flashes. Active markers can be realized with the help of LED lights, for example. Coding of the markers allows point numbers to be assigned to the object points in the evaluation. In the field of augmented reality, ArUco markers are widely used for this purpose [16], for example.

An essential aspect in photogrammetric image processing is the *accurate placement of markers in an image*. The simplest approach is segmentation by threshold analysis [17]. Here, the input image or a section of the image is binarized by applying a threshold value to its gray values. The segmented image regions are further processed by applying knowledge about the appearance of the marker (size or shape, for example). A prerequisite for this procedure is a sufficient contrast between foreground and background of the marker. The advantage of threshold analysis, apart from fast processing, is that a wide variety of shapes can be segmented by analyzing different properties of the connected components of the segmented regions. For a high precision, subpixel accurate edges are typically extracted after the marker has been segmented. This allows geometric primitives to be fitted to the previously segmented markers, such as ellipses for circular markers. These primitives allow the markers to be determined with high accuracy. A common operator for extracting gray value edges is the *Canny-Operator* [18]. According to [4], this is characterized by high sensitivity to the true edge, immunity to noise, and high accuracy of the edge position. The *Deriche-Operator*, according to [19], is a further development of the *Canny-Operator* and operates with a recursive filter [4]. Especially for circular markers, the Hough Transformation [20] is also a way to segment and measure the marks: image points are transformed into a dual space that is parameterized depending on the geometric primitive. A robust and scale-invariant approach to the size for circular markers is presented in [21]. Drawbacks of the Hough transform are the computational intensity of the algorithm due to the large number of all possible transformations and the difficulty of determining a suitable threshold for the votes in the dual space [22].

Another aspect to be considered in *object recognition* is the object point mapping or correspondence analysis of 2D image to 3D object points. For this reason, coded markers are preferred in industry. If, on the other hand, non-coded markers are used, correspondence analysis is necessary. One method for this is the random sample consensus (RANSAC) [23]. The algorithm selects random samples from the set of 2D image points, along with 3D object points, and forms hypothetical correspondences. From these correspondences, a pose can be computed (e.g., by applying a PnP algorithm). To evaluate the quality of the pose, the remaining 3D object points are projected back into the image using the pose and compared to the position of the extracted image points. Many RANSAC implementations work with feature points and optical flow. In contrast to the approach of this work, a pose of the stereo camera system or the object is calculated over a large set of feature points using RANSAC for pose estimation [24,25]. Another method for solving the correspondence problem is “simultaneous pose and correspondence determination” [26] and its extension [27]. This algorithm does not only use subsets of hypothesized correspondences to find the optimal pose of the object, but all available image and object points are used simultaneously for iterative correspondence analysis. Disadvantages of the method are its long run time and the high sensitivity of the algorithm to the input parameters.

When using a stereo camera system, object recognition also includes the determination of the same object point in the two stereo images, i.e., a correspondence analysis between image points. Exploiting the epipolar geometry is beneficial for this correspondence search. If the orientation is known, stereo images can be converted to the stereo normal case and correspondences can be found robustly and efficiently [17].

## 2. Methods

In this chapter, we present the developed methods of the prototype. For this purpose, we introduce the used mathematical camera model, which provides the basis for the final estimation of the object pose. Subsequently, the algorithms developed for object point detection and pose estimation are presented.

### 2.1. Camera Model

The mathematical abstraction of the camera system is implemented by using a pinhole camera model. The 3D object points of a calibration object are assumed to be known, and their corresponding 2D image points are measured in the images. Then, the camera calibration can be written as the minimization of the following error function [17]:

(1) $$d\left(\Theta \right)=\sum_{l=1}^{2}\sum_{k=1}^{n_{o}}\sum_{j=1}^{n_{m}}v_{j,k,l}{∥\pi_{r}\left(m_{j,k,l},\Theta_{r,l}\right)-\pi_{i}(M_{j},\Theta_{i,l})∥}^{2}⟶\min.$$

This operator minimizes the differences between the 3D object points of the calibration object and the rectified image points on the image plane. Rectified pixels are characterized by the elimination of geometric distortion. The number of markers $n_{m}$ corresponds to the number of object points. The number of object poses is defined via $n_{o}$. Note that for the calibration, $n_{o}$ corresponds to the number of acquired calibration images of the calibration object. Later, for real-time pose estimation with the prototype system (see Section 2.3), $n_{o}$ is 1 because only one stereo image pair is taken of the machine for pose determination. The sum with the index *l* is used to model the stereo case. For a monocular camera system, the sum is omitted accordingly. The 3D positions of the object points of the calibration object are represented in M. Correspondingly, the projections of the object points onto the image are denoted by m. The vector Θ contains the interior and exterior orientation parameters of the camera model. The parameters of the interior orientation are the focal length (*f*), the pixel size ($s_{x}$ and $s_{y}$), the principal point ($c_{x}$ and $c_{y}$), and the lens distortion [17]. Firstly, the simple division model (κ) [17] was used for distortion modeling in the simulations, and secondly, the Brown distortion model ($K_{1}$, $K_{2}$, $K_{3}$, $P_{1}$, and $P_{2}$) [28] was used for the prototype. For the simulation, the division model was used to keep the complexity of the simulation model low. With the transformation $\pi_{r}$, the projection of the 3D object points onto the undistorted image plane was calculated. On the other hand, $\pi_{i}$ is the rectification of the (distorted) image points onto the undistorted image plane. Thus, the lens distortion is eliminated. Hence, the difference between the results of the two transformations $\pi_{r}$ and $\pi_{i}$ is minimized by the model. The variable vs. indicates whether the object point is visible in the current image or not, and is therefore 1 or 0. Details about the used camera model can be found in [17].

### 2.2. Object Point Detection

**Figure 1:**
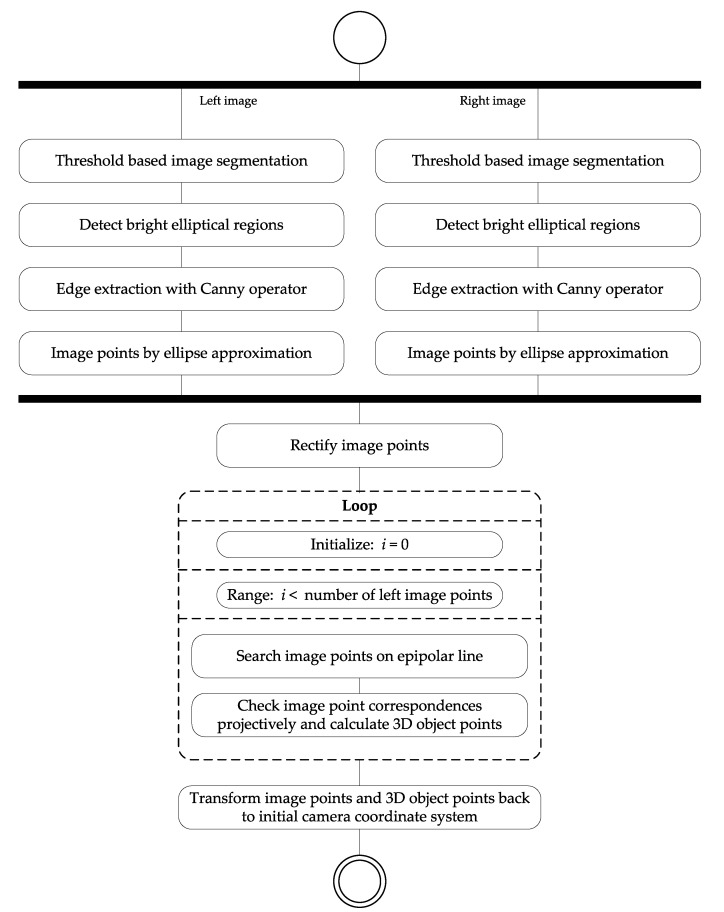
Activity diagram of the image processing for segmentation of the object points in the images and correspondence analysis among the segmented image points in the stereo image pair.

Our algorithm to detect and measure circular object points (see LED markers in Section 4.1) in the stereo image pairs is explained below. Input information is a calibrated stereo image pair, and output information is the corresponding image points of the detected object points and their reconstructed 3D coordinates in the camera coordinate system. The entire processing is shown in Figure 1.

**Figure 2:**
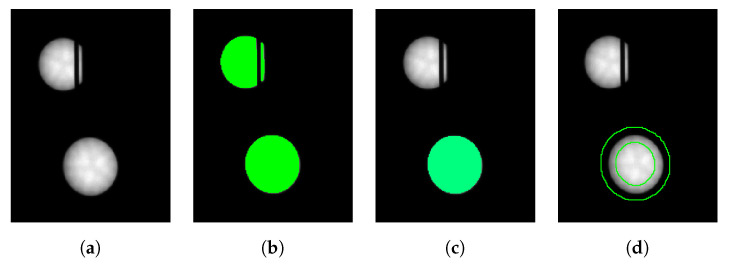
Steps of segmentation of the object points in the image visualized on a cropped gray value image part (**a**). While during threshold segmentation (**b**), interrupted LED markers are still detected, they are eliminated when applying the selection criteria (**c**). Morphology is used to determine the edge regions of the LED markers (**d**).

The first step is to segment and measure the projections of the object points in the two images. With the requirement that the system is used in dark environments and due to the large aperture number, LEDs are imaged with high contrast (see Figure 2a). Thus, it is possible to use a simple and fast thresholding method for the first segmentation of the image. The threshold value is calculated dynamically using the gray value histogram of the image. For this purpose, the first local minimum of the histogram after a Gaussian smoothing with σ=4 gray values is defined as the threshold value (see Figure 2b). To detect circular LED markers in the image, all connected components of the segmentation are examined for their circularity $C^{\prime }$, compactness $C^{\prime \prime }$, and area [29]. For an optimal circle, $C^{\prime }$ and $C^{\prime \prime }$ parameters are equal to 1:

(2) $$C^{\prime }=\frac{F}{\max{\left(r\right)}^{2}\;\cdot \;\pi }\;,\;C^{\prime \prime }=\frac{L^{2}}{4\;\cdot \;F\;\cdot \;\pi }\;.$$

Here, *F* is the area of the segmented region, *L* is the length of its contour, and *r* is the maximum distance from the center to all contour pixels. The area can be restricted based on the knowledge about the size, perspective, and camera distance of the object points. Note that because elliptical regions are to be found, the thresholds for $C^{\prime }$ and $C^{\prime \prime }$ must be relaxed accordingly (see Section 4.1). Although in all our experiments (see below) the circularity measure was sufficient, an ellipticity measure [30] could be used instead, which would increase the robustness even further. The result after applying the three criteria is shown in Figure 2c. In the next step, the selected elliptical regions are prepared for edge extraction. For this purpose, the area of transition from the dark background to the light marker is selected via region morphology [17]. The difference region between the dilated and eroded marker region forms the ROI for subpixel edge extraction by using the Canny operator [18] (see Figure 2d). After pixel-precise edge extraction, we apply a non-maximum suppression to the edge amplitude in gradient direction and skeletonize the result. Finally, subpixel-accurate edge extraction is performed and the resulting edge points are linked to subpixel-precise contours. The process of edge extraction is described in detail in [17]. Other parameters are also conceivable for region-based segmentation, depending on the geometric shapes of the markers used. For example, the rectangularity [29] or the length of the contour.

**Figure 3:**
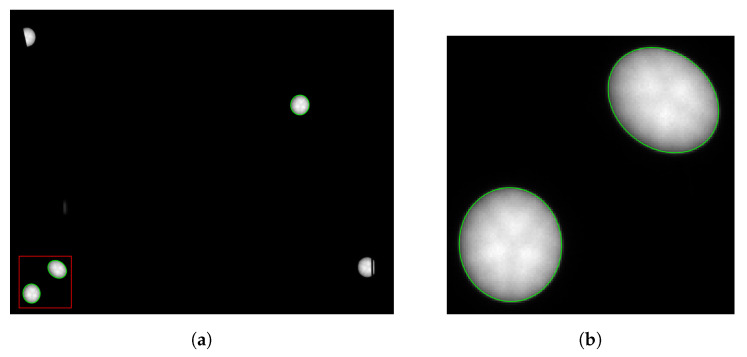
Result of object points segmented by ellipses (green) in the image (**a**). Non-unique and interrupted object points are automatically eliminated. (**b**) A detailed enlargement.

Then, ellipses are fitted to the extracted edges by minimizing the squared algebraic distances between the edge points and the ellipse according to [17]. Outliers in the edge points are eliminated by Tukey’s weight function [31]. The result of the ellipse calculation is visualized in Figure 3a. The figure also shows LED markers that are partially occluded and are eliminated by the criteria from Equation (2) in order to avoid inaccuracies. The coordinates of the image points of the LED markers are the centers of the fitted ellipses. Note that because we use a sufficient number of marks, we can afford to simply eliminate occluded or disturbed marks instead of trying to accurately determine their centers. Having the practical application in mind, we prefer robustness and accuracy over redundancy in this case.

**Figure 4:**
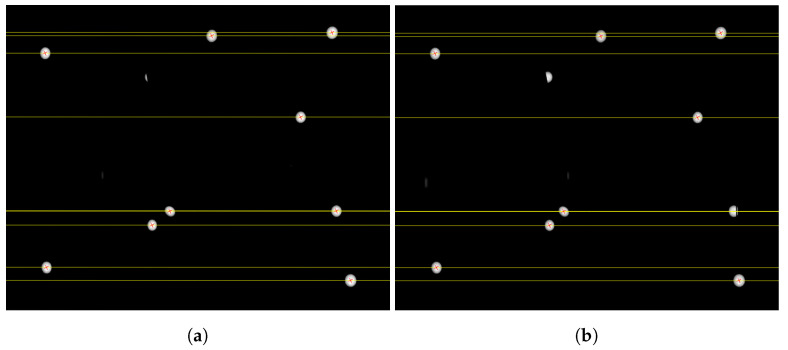
Epipolar lines (yellow) in the left (**a**) and right (**b**) rectified images of the stereo normal case with the found image point correspondences (red).

Among the image points found in the left and right stereo images, point correspondences must be established to perform a stereo reconstruction. For this, the epipolar geometry is exploited. After calibrating the stereo camera system, the two stereo images are rectified into the stereo normal case [17]. Corresponding image points in the left and right stereo image lie in the same image line (see Figure 4). Furthermore, lens distortions in the rectified images are eliminated. For each image point of a marker in the left stereo image, it is checked whether an image point of a marker exists on the corresponding epipolar line in the right stereo image. The threshold value ($\sigma_{epi}$) for the check is determined depending on the calibration of the stereo camera system. If points are found on the epipolar line, it is checked whether the optical rays from the left and right image intersect in the object space in front of the stereo camera system. If successful, an image point correspondence is found and the 3D point is calculated. Finally, the image points and the 3D coordinates of the object points are transformed back from the rectified coordinate system of the stereo normal case into the original coordinate system.

### 2.3. Object Pose Estimation

**Figure 5:**
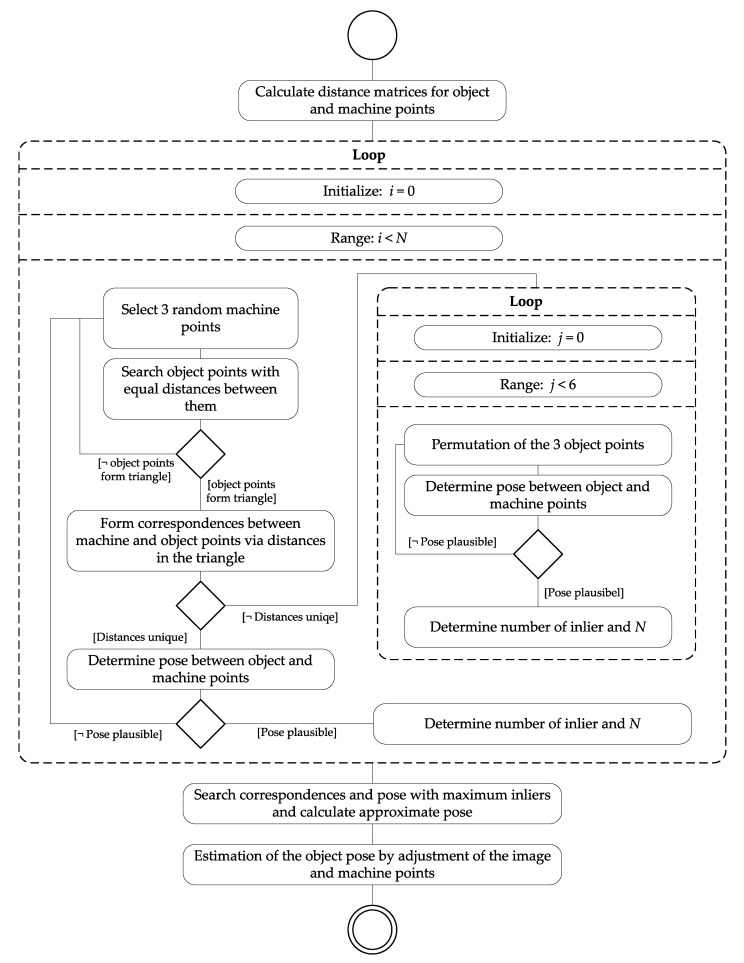
Activity diagram of the correspondence analysis between 3D machine and 3D object points using the RANSAC approach and subsequent calculation of the object pose.

To calculate the object pose, a correspondence analysis between the detected 3D object points and 3D machine points is necessary. The machine points indicate the positions of the object points in the machine system. They can be measured once beforehand, for example, with a robotic total station, a laser tracker, or by the stereo camera system itself. The RANSAC-based algorithm for point correspondence analysis and estimation of the object pose is visualized in Figure 5 (explained in the following text).

We exploit the knowledge about the geometric configuration of the machine points to increase the reliability and performance of RANSAC. The distances between the points are used during point selection of machine and object points for hypothetical correspondence formation in RANSAC. A matrix with the euclidean distances is calculated for each pair of machine points. To increase the correspondence search, the relative distances of the object points are sorted in a list with the corresponding point numbers.

A termination criterion is set for RANSAC, which represents the maximum number of iterations *N*. In [32], *N* is defined as follows:

(3) $$N=\frac{\log\left(1-p\right)}{\log\left(1-{\left(1-\epsilon \right)}^{s}\right)}.$$

Here, s=3 is the minimum number of data points needed to compute the model, *e* is the proportion of data expected to be outliers, and p=99.9% is the required probability of success. The proportion of outliers *e* is initialized with 1.0 and updated dynamically after each iteration. Subsequently, *N* is updated according to Equation (3).

For hypothetical correspondence formation in RANSAC, three different machine points are randomly selected. The three distances between the selected points are looked up in the distance matrix of the machine points. From the sorted list of object point pairs, point pairs with identical distances (up to a tolerance threshold) are selected. The tolerance threshold depends on the system accuracy and is in the lower centimeter range. Due to noise, it is possible that more than three corresponding distances and thus more than three corresponding object points will be found. Therefore, it is checked which three object points form a closed path and thus a triangle over the extracted distances. Before the correspondence analysis, it is checked for each set whether the distances in the triangle of object points are unique and can be unambiguously assigned to the distances in the triangle of machine points. If this is the case, direct formation of hypothetical point correspondences between machine and object points is possible for the respective sets. Otherwise, all six permutations are tested. For each set of three corresponding points, the pose of the machine points with respect to the object points is calculated, by reducing the distances between them using a 3D rigid transformation with 6 parameters [29]. The pose is checked for plausibility, e.g., whether the machine is standing upright. For all plausible poses, the number of inliers is determined by checking the 3D distances between transformed machine points and object points. The threshold value $d_{trans}$ depends on the system accuracies and linearly on the distance between the camera system and object.

Finally, the pose is selected that resulted in most inliers. If there are several equivalent poses, the pose is selected in which the sum of the distances of the corresponding points is the smallest. The pose is re-computed by using all inliers. This pose serves as an approximate pose for a subsequent least squares adjustment where the error in the original observations, i.e., the image points, is minimized according to Equation (1). For the calculation, all camera parameters of the interior and relative orientation are kept fixed and only the object pose is estimated. Interior and relative orientation of the stereo camera system are set to the previously calibrated values. The observations are the image points of the object points, and the 3D machine points are assumed to be known. All observations are equally weighted in the adjustment. The result of the adjustment is the final pose of the machine in the camera coordinate system.

## 3. System Selection

The prototype system was designed by calculating and evaluating simulations. For the design, it was assumed that the machines to be measured will be so large that markers can be attached to their rear sides. The camera system should have an operating range of up to 50 m. A Monte Carlo simulation was used to determine the optimum design of the prototype camera system. Later, the selected camera system is described in detail.

### 3.1. Influencing Aspects and Simulations

Various influencing factors have an effect on the performance of the final system. The influences were determined and quantified in various simulations to gain the optimum system.

The first investigated influence was the configuration of the object points, including the spanned volume, the number, and the arrangement of object points. The volume ranged from 1 m×1 m×0.4 m to 2 m×1.5 m×0.6 m, which represents possible mounting options at the rear of a construction machine. The number of object points ranged from 5 to 13, as 5 object points covers the minimum requirement for robustness against occlusions, whereas more than 13 would be too elaborate to be mounted on the machine. A further distinction in the simulations was made between the camera configurations. On the one hand, a monocular camera system was simulated, and on the other hand, a stereo camera system with different bases. The disadvantage of the monocular camera system is the limited accuracy of the depth estimate of the pose, especially for larger focal lengths. Therefore, additional stereo camera systems with two different bases were simulated. A base of 0.5 m allows a compact stereo camera system, whereas a base of 8 m provides a better depth estimate, but the handling of the 8 m base system is more difficult in demanding environments.

Furthermore, three different camera sensors were simulated for each of the three camera configurations: a 1/1.2${}^{\prime \prime }$ sensor with 2.35 MP, a 1.1${}^{\prime \prime }$ sensor with 20.4 MP, and a APS-C sensor with 31.4 MP.

As a further impact, the distance between the camera system and the object was taken into account. For the simulations, discrete distances of 5 m, 25 m, and 50 m were assumed for this purpose. To cover the large distance range, it is essential to use a lens with an appropriate focal length. For this purpose, the three focal lengths of 10 m, 25 m, and 50 mm were investigated in the simulations.

All simulations were performed by using the Monte Carlo method [33]. The accuracy estimation was carried out according to the random principle with a large number of scattering observations as input variables. We set the sample size to 100,000 runs. The advantage of the Monte Carlo method in contrast to the classical variance propagation is that no partial derivatives have to be known [34].

**Figure 6:**
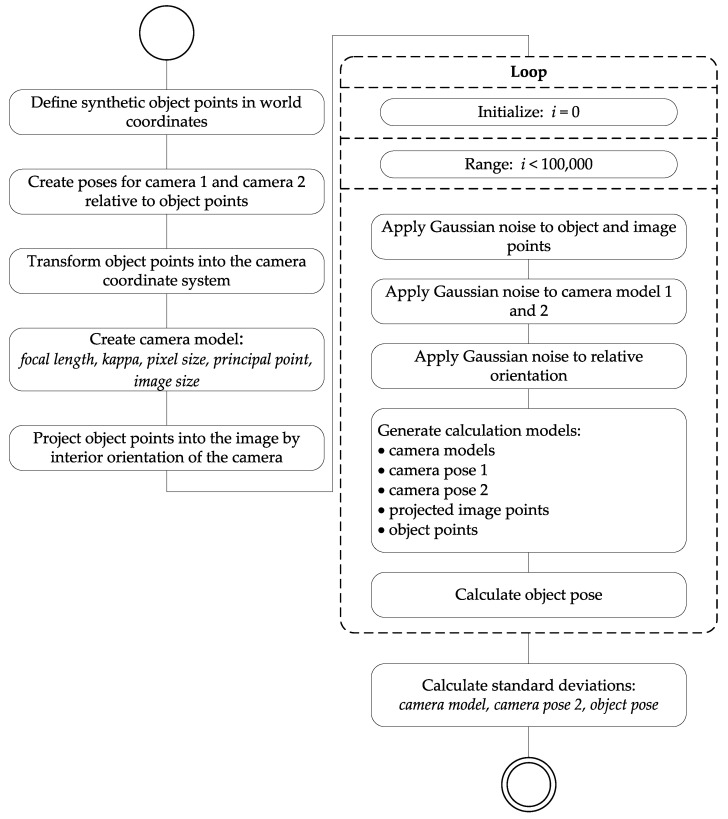
Activity diagram of the Monte Carlo simulation for accuracy estimation of the overall system using the example of the stereo camera system.

The Monte Carlo simulation procedure for the stereo case is outlined in Figure 6. In preparation, synthetic image points had to be created from the 3D object points. The camera poses were chosen such that the optical axes of the cameras pointed to the center of the object points. With the poses, the 3D object points were transformed from the world coordinate system into the camera coordinate system via a 3D rigid transformation with 6 parameters. Then, the transformed 3D points were projected into the image by using the interior orientation of the camera. Gaussian noise with different standard deviations was applied to the 3D points in the world coordinate system and the image points in the pixel coordinate system (see Table 1). In a first step, the parameters of the interior orientation were assumed to be error-free. To estimate the influence of the camera calibration on the result, it was necessary to also apply noise to the parameters of the interior orientation(s) and to the parameters of the relative orientation in the stereo case. This was done in a second simulation step. With the input parameters and the previously calculated pose as an approximation, the object pose was calculated 100,000 times according to Equation (1).

**Figure 7:**
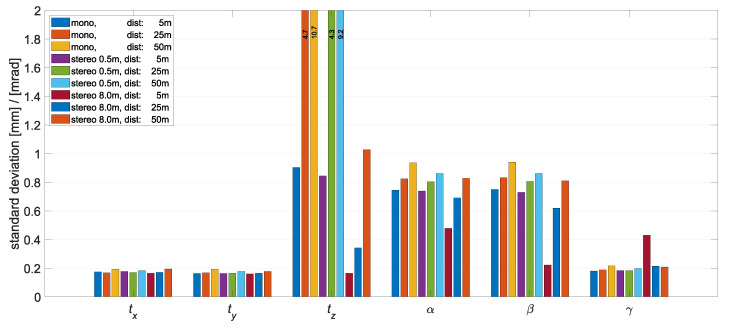
Standard deviations of the object pose from the Monte Carlo simulation over the different recording configurations. Simulated was the 20.4 MP sensor, with a focal length of 25 mm, and a 2 m×1.5 m×0.5 m object.

First, the results for an error-free interior orientation are discussed. The reference system is the camera coordinate system. Figure 7 visualizes the standard deviations of the resulting object pose for the 20.4 MP sensor, with a focal length of 25 mm, detecting a 2 m×1.5 m×0.5 m object. The accuracies of the stereo camera system that affect the depth estimation are better than those of the monocular camera system. These are the translation $t_{z}$ and the rotations α and β, which in principle expresses the expected advantage of the stereo system. The smaller the distance-to-base ratio, the better the intersection angle of the optical rays of the two cameras and the smaller the uncertainty in the depth reconstruction of a 3D point. In the two rotation components α and β, a significant improvement was observed at close range. This is related to the size of the distribution of the object points in the z-direction. This is because a large difference in the z-components of the 3D points brings better stability in α and β.

**Figure 8:**
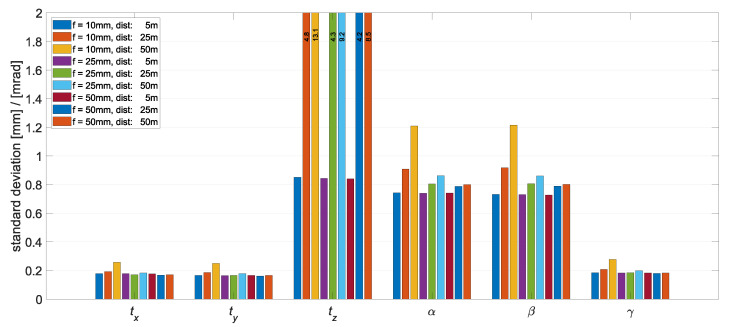
Standard deviations of the object pose from the Monte Carlo simulations with different focal lengths. Simulated was the 20.4 MP sensor, a stereo base of 0.5 m, and a 2 m×1.5 m×0.5 m object.

Figure 8 shows the results for different focal lengths and for a stereo camera system with a base of 0.5 m. The camera sensor and the object point arrangement were chosen as in the previous experiment. On all six parameters of the object pose, it can be seen that the influence of the focal length is similar. The simulations with a focal length of f=10 mm resulted in the largest standard deviations in all components. Comparing the focal lengths of 50 and 25 mm, the larger focal length shows significant improvements for long-range detection, especially in the components $t_{z}$, α, and β, where the depth estimate has an influence. This shows that the size of the area onto which the object points are projected in the image plane influences the accuracy of the object pose. For all other simulations with different object point arrangements, camera configurations, camera sensors, and focal lengths, equivalent results were obtained. In general, in all four previously discussed analyses, it can be seen that the uncertainties in all components of the object pose also increase with increasing distance between the camera system and the object.

In a second step, the influences of the accuracy of the camera calibration on the uncertainties of the object pose was evaluated. For this purpose, the different results that were obtained with error-free camera parameters are investigated. The standard deviations in $t_{x}$ and $t_{y}$ approximately increased by 0.5 mm, which corresponds to an increase of approximately 15% to 30%. In $t_{z}$, the absolute increases are larger and also increase with increasing distance between the camera system and the object. The relative increase is smaller and ranges from 5% to 20%. The increase in the rotational components is in the thousandths of a millirad and thus negligible. In some simulation setups, the uncertainty of the distortion κ has a large influence on the parameters $t_{x}$, $t_{z}$, and β, because of geometric conditions. All in all, the results show that good camera calibration is indispensable for the use of a camera system over long distances.

### 3.2. Camera System and Calibration

The simulations show that the best results were achieved with a stereo camera system with a base of 8.0 m, a 20.4 MP image sensor, and a focal length of 50 mm. However, the simulations also show that the accuracy of a stereo camera system with a base of 0.5 m was sufficient. Therefore, the more compact system was chosen because it has significantly greater mobility. A 10 mm steel plate was used as the base plate, on which the cameras were mounted. Two Lucid Triton 24.5 MP monochrome industrial cameras were chosen as camera sensors. They are equipped with the Sony IMX540 $4/3^{\prime \prime }$ CMOS sensor with a resolution of 5320 × 4600 pixels and a sensor element size of 2.74 μm. Due to the monochrome gray scale model, the sensor delivers full resolution, and no inaccuracies in the image measurement will be created by color filters. With 4.9 fps, the camera is able to deliver a sufficiently high frame rate to realize a real-time version of the system. The camera was controlled via the GigE Vision standard [37]. The two cameras were synchronized via the Precision Time Protocol (PTP) [38]. For the transmission of the image information of an 8 Bit image in full resolution via the Gigabit Ethernet interface, a camera needs approximately 220 ms. With a short time delay between the data packets and a short exposure time of the images, a triggering and transmission of the stereo image pairs with 2 Hz is technically possible.

The choice of the focal length depends on the size of the field of view of the camera system in the object space and on the size of a marker in the image. According to [4], the accuracy of the point measurement decreases exponentially starting from an imaging marker diameter of 15 pixels. However, a minimum width of the FOV is required to localize the machines over a wide operating area. For our application, we assume a FOV of 10 m. The evaluation was carried out for focal lengths of f=25 mm and 50 mm, with a sensor element size of s=2.74 μm, and for a marker diameter d=66 mm over the distance *D* using the following formula:

(4) $$\#\mathrm{pixel}=\frac{d\;\cdot \;f\;\cdot \;s}{D},\;\mathrm{FOV}=\frac{D\;\cdot \;s\;\cdot \;5320\mathrm{pixel}}{f}.$$

**Figure 9:**
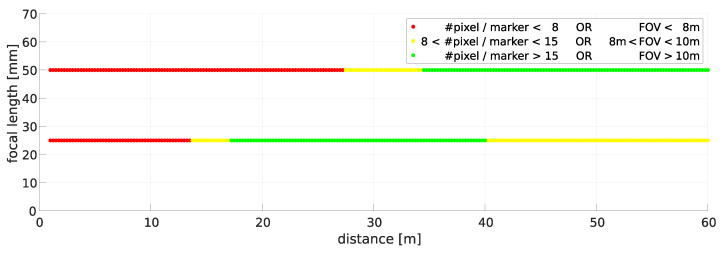
Operating range of different focal lengths with the Sony IMX540 as a function of the field of view and the number of imaged pixels in the image for a 66 mm sized marker.

The results of the evaluation are visualized in Figure 9. In the close-range, the FOV is the limiting factor, whereas in the far-range it is the number of imaged pixels of a marker. With a focal length of 25 mm, a range from 20 m to 40 m, and a focal length of 50 mm, a range from 40 m to 60 m can be covered. Based on the result, the stereo camera system of the prototype was equipped with 25 mm lenses. A Fujinon CF25ZA-1S lens for $4/3^{\prime \prime }$ sensors with an aperture range of F1.8–F16 was selected for this purpose.

Due to the large variations of object distances, a large depth of field is necessary. According to [4], an aperture stop of 5.6 and a focused object distance of 10 m results in a depth of field from 5.4 m to 72.0 m. Although a higher f-number would further extend the depth of field, the exposure time would have to be increased as well, which would lead to motion blur for moving objects. Additionally, higher f-numbers cause diffraction effects of the light at the aperture, which in turn lead to blurred images [17].

**Figure 10:**
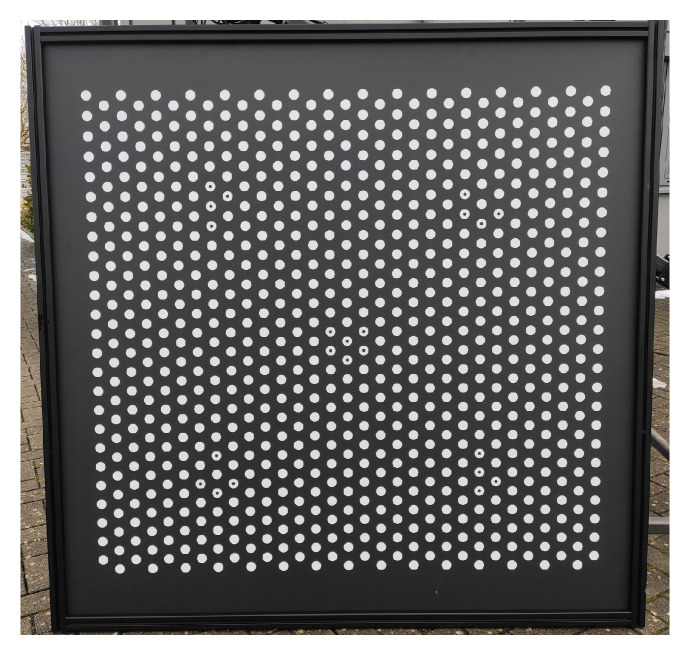
Planar calibration object with a size of 1.5 m×1.5 m and 792 hexagonally arranged circles.

High-precision measurements with camera systems require careful calibration of the system in advance. This includes determining the interior orientation and relative orientation. The left stereo camera defines the origin of the camera coordinate system. The translation and rotation of the right stereo camera define the base of the stereo camera system. Mathematically, the calibration was implemented via simultaneous calibration with bundle adjustment by Equation (1). For this purpose, several images were acquired of a planar HALCON calibration object with an extension of 1.5 m×1.5 m and 792 hexagonally arranged circles (see Figure 10) [17].

The rectified image points of the measured calibration marks were used for the adjustment. During calibration, their distances to the projected circle centers of the calibration object were minimized. Lens distortions were modeled with the polynomial distortion model [17], which contains three parameters for the radial and two parameters for the decentering distortions. This model is based on the work of [28,39]:

(5) $$\begin{array}{cccc} & \; & \left(\begin{array}{c} u\\ vs. \end{array}\right) & =\left(\begin{array}{c} \tilde{u}\left(1+K_{1}{\tilde{r}}^{2}+K_{2}{\tilde{r}}^{4}+K_{3}{\tilde{r}}^{6}\right)+P_{1}\left({\tilde{r}}^{2}+2{\tilde{u}}^{2}\right)+2P_{2}\tilde{u}\tilde{v}\\ \tilde{v}\left(1+K_{1}{\tilde{r}}^{2}+K_{2}{\tilde{r}}^{4}+K_{3}{\tilde{r}}^{6}\right)+2P_{1}\tilde{u}\tilde{v}+P_{2}\left({\tilde{r}}^{2}+2{\tilde{v}}^{2}\right) \end{array}\right), \end{array}$$

with ${\tilde{r}}^{2}={\tilde{u}}^{2}+{\tilde{v}}^{2}$.

After the system calibration, the distances of all corresponding image points of the calibration marks in the left and right stereo image to the respective epipolar lines were determined. The standard deviation $\sigma_{epi}$ of the distances was calculated for all image point correspondences and used as an evaluation metric in the object point detection algorithm.

## 4. Evaluation

The first part of the evaluation was the field measurements, which tested the performance of the selected system and the developed algorithms. For this purpose, the signalization of the object points is described first, which resulted in the threshold values for the generically developed algorithms. Subsequently, the structure of the test environment is described, along with the reference measurement in a superordinate reference frame. The second part of the evaluation was the analysis of the accuracies achieved with the prototype system within the field measurements.

### 4.1. Field Measurements

**Figure 11:**
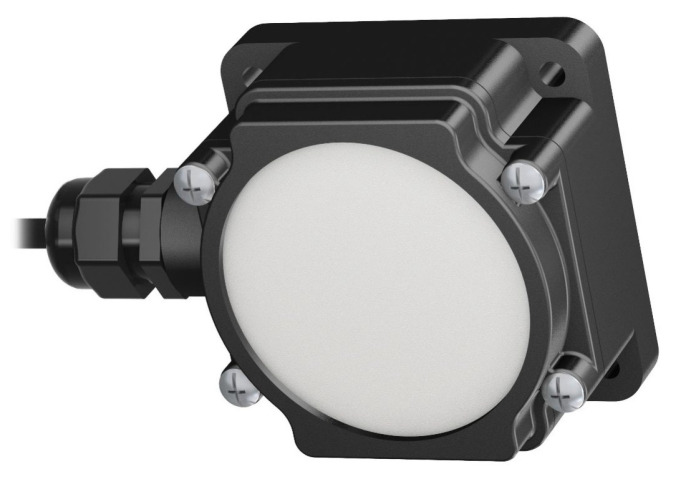
LED light indicator as active marker for object point signaling. Image according to [41].

For object point signaling, active markers are used due to the possible poor ambient conditions on construction sites. In addition, active markers allow measurements even during the night. The quantum efficiency according to EMVA 1288 [40] of the used Sony IMX540 image sensor must also be taken into account. This is highest in the wavelength range that includes blue and green. Ten industrial LED indicators are thus used for object point signaling in the prototype (see Figure 11). They are IP-67-certified and have low power consumption due to the LED technology. The lamps have a circular diffuse green light area with a diameter of 66 mm. The circular shape allows accurate measurements of the centers of the markers in the image. To minimize interfering light of other spectral colors from the environment in the image, a band-pass filter for the wavelength range from 500 nm to 555 nm is applied to the cameras.

Due to the central perspective mapping in the pinhole model, circular objects are imaged as ellipses [42] (when ignoring lens distortions). In general, the centers of the ellipses in the image deviate from the imaged circle centers by an eccentricity. With increasing diameter of the marker, increasing angle between image plane and object circle plane, and increasing distance between marker and optical axis, the influence of the eccentricity increases. For high-precision measurements, this must be taken into account [42]. For the use of the prototype, the influence of the eccentricity is estimated with the parameters of the previously selected camera system and marker. As a result, the eccentricity is smaller than 0.5 μm from a distance of 9.5 m, and thus negligible according to [4].

The selection of the markers and the camera system resulted in the threshold values for the object point detection algorithm (see Equation (2)). Based on knowledge about the size, perspective, and camera distance of the object points, the area *F* can be restricted, for example, from 30 pixels to 7000 pixels, in our application. The parameter $C^{\prime }$ was empirical restricted from 0.5 to 1.0 and $C^{\prime \prime }$ from 1.00 to 1.15 (see Section 2.2). In order to perform stereo reconstruction, point correspondences must be established. For the described epipolar evaluation, the threshold value $6\sigma_{epi}$ has to be defined. As a result of the calibration of the stereo camera system, it was 2.51 pixels in our application.

A threshold value must also be defined for the object pose estimation algorithm. The parameter $d_{trans}$ limits the 3D distances between the transformed machine points and object points. In our case, it was set to 25 mm at 10 m and 65 mm at 50 m.

#### 4.1.1. Experimental Setup

**Figure 12:**
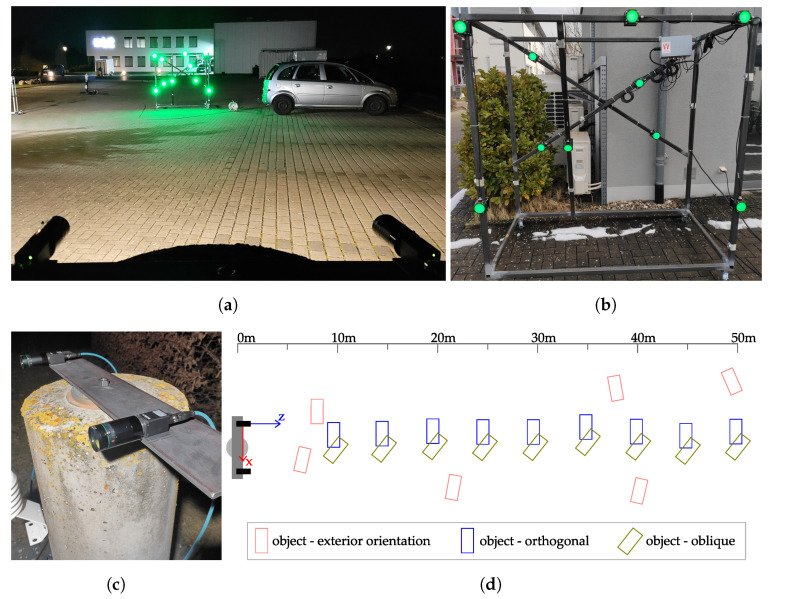
Overview of the experimental setup (**a**) with the object to measure (**b**) and the measuring pillar with the stereo camera system (**c**). A sketch shows the test setup with visualization of the camera coordinate system and the various positions of the object (**d**).

To test the system, we created a test environment that represents the requirements as realistically as possible (see Figure 12a). This included a dark environment with sources of interfering light. A steel frame was used to simulate the machine. The LED markers were attached to the steel frame in a volume of 2 m×1.5 m×1 m (see Figure 12b). This simulated the possible spatial distribution of the markers on a machine. In the foremost plane to the stereo camera system, five LEDs were distributed over an area of 2 m×1.5 m. Offset from the plane one meter in parallel, four LEDs were mounted, and one LED marker was mounted between the two planes. During mounting, care was taken to ensure that the markers were not arranged symmetrically to avoid ambiguities in the rotations of the object pose and in the relative distances. The stereo camera system was mounted on a measurement pillar (see Figure 12c) so that no movements in the camera system were to be expected during measurement series. The exterior orientation of the stereo camera system with respect to a reference system was determined by a spatial resection by measuring the object at six different positions (see Figure 12d).

A schematic overview of the measurement setup is shown in Figure 12d. The origin of the camera coordinate system is in the optical center of the left stereo camera. The *z*-axis points along the optical axis into the object space and the *x*-axis in the direction of the right stereo camera. The *y* axis completes the right-handed coordinate system. This coordinate system was used to evaluate the measurement series. The object was measured at six different positions to determine the exterior orientation of the stereo camera system in the local reference frame spanned by a laser tracker. These were distributed up to 8 m to the right and 3 m to the left of the optical axis at a distance of 6 m to 50 m, and thus covered the entire measurement area. The object was measured at 5 m intervals at distances from 10 m to 50 m from the stereo camera system. At each distance, the object was aligned once orthogonally and once obliquely to the optical axis of the stereo camera system. The rotation about the *y*-axis was 25° to 45°. Six stereo image pairs were acquired for each position of the object to be able to calculate the standard deviations.

The stereo camera system was calibrated with 72 stereo image pairs. The root mean square error (RMSE) of the back projected image points was 0.26 pixel.

**Figure 13:**
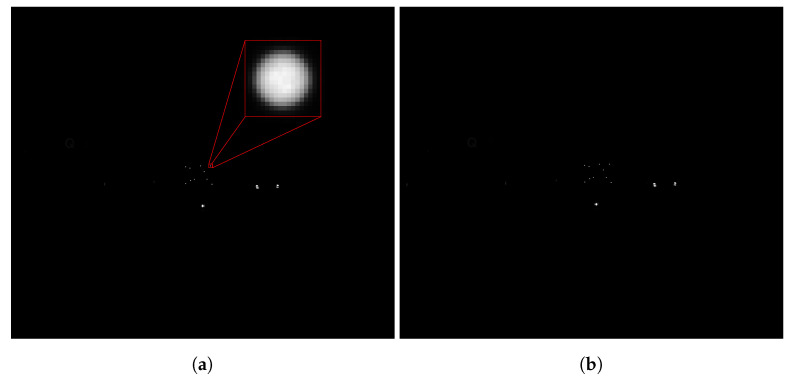
Left (**a**) and right (**b**) images of a stereo image pair of the test measurement. Object at 45 m distance with additional interfering light in the scene. A zoom (red) shows the original resolution of an imaged LED marker.

Figure 13 shows one exemplary stereo image pair of the camera system. The exposure time for all images of the entire measurement series was 2 ms.

#### 4.1.2. Reference Measurement

A reference system should provide ten times the accuracy of the system under investigation. The stereo camera system was expected to have accuracies in the millimeter to centimeter range. The reference system therefore requires a measurement accuracy in the sub-millimeter range. This was achieved by using the Leica Laser Tracker AT402 [43].

The positions of the LED markers on the object were determined with the laser tracker by measuring in the machine coordinate system. For this purpose, the edge of the illuminated surface of the LED marker was measured at at least five positions with the laser tracker. All measurements approximated a circle that defines the center of the illuminated surface, and thus the marker coordinate of the LED marker in the machine system. In addition, nine reference points were attached to the object. The positions of these in relation to the ten LED markers were determined as part of the calibration. Later in the evaluation, it was thus possible to precisely measure the position of the object via the reference points.

The determination of the pose of the coordinate systems of the stereo camera system with respect to the laser tracker was done by positioning the object at the six different locations (see Figure 12d). For this purpose, the reference points of the object were measured at each position. Using a 3D rigid transformation with six parameters, the coordinates of the LED markers from the calibration were transformed to the corresponding positions via the measurements of the reference points. Thus, the coordinates of the LED markers were determined in the system of the laser tracker. The extraction of the image coordinates of the LED markers was done via the algorithm presented before. The image coordinates for each LED marker were averaged from the six stereo images at each of the six locations. The calculation of the 3D transformation between the camera coordinate system and the coordinate system of the laser tracker was performed via an adjustment using function Equation (1). In total, 59 3D coordinates of the LED markers in the laser tracker system and 118 image coordinates of the stereo camera system were included in the adjustment. All parameters of the interior and relative orientation of the stereo camera system were kept fixed during optimization, so that only the pose between the object and the stereo camera system was estimated. The pose that was estimated describes the transformation between the two coordinate systems and thus the exterior orientation of the stereo camera system. The RMSE of the adjustment was 1.13 pixel.

The determination of the reference pose of the object required the determination of the LED markers with the laser tracker at each position where the object was measured with the stereo camera system. As before, the marker coordinates of the LED markers were determined by means of a 3D rigid transformation with six parameters via the measurement of the reference points from the calibration and the current position of the object. The reference pose of the object was calculated by transforming the coordinates of the LED markers in the machine system from the calibration to the previously determined marker coordinates of the LED markers in the camera coordinate system from the laser tracker measurement. This process determines the reference pose of the object in the submillimeter range.

### 4.2. Accuracy Evaluation

In this section, the field measurements are evaluated to assess the relative and absolute accuracy of the system.

#### 4.2.1. Relative Accuracy and Repeatability

A quality criterion for analyzing the systems precision is its repeatability. This indicates the variation of the measured values around the mean value and is calculated as follows:

(6) $$\sigma_{rpt}=\sqrt{\frac{\sum_{i}^{n}{\left(\bar{x}-x_{i}\right)}^{2}}{n-1}}.$$

To evaluate the precision of the object poses, the mean pose was calculated from the six calculated object poses at each position. The component-wise difference formation to the mean value resulted in six repeatability standard deviations of the system.

**Figure 14:**
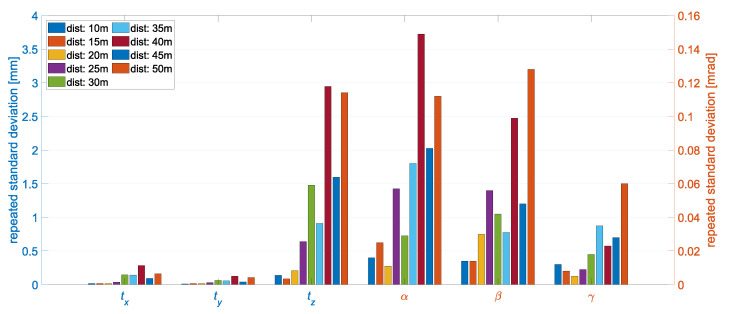
The systems precision is represented by the repeated standard deviations of the object pose from the test measurements at distances varying from 10 m to 50 m. Object orientation: orthogonal.

The standard deviations of the object poses from the measurement series are visualized in Figure 14 for the orthogonally aligned positions. Overall, the repeated standard deviations were in the millimeter or submillimeter range. In the translations $t_{x}$ and $t_{y}$, the values are even smaller than 0.3 mm. The largest variation was observed in $t_{z}$. This is typical for a stereo camera system and is to be expected from the results of the previous simulations. This distance dependence can be seen in all six pose components. Of the rotations, γ was the rotation with the lowest repeated standard deviation. The previous simulations showed that this resulted from the fact that the depth estimate does not have a significant influence on the rotation γ. At the distance of 40 m, inaccuracies in the input data from the measurement series are to be assumed. As in comparison with the neighboring distances, the results were not true to expectations. The previous simulations do not show this behavior.

For the positions where the object was oriented obliquely to the stereo camera system, the results show the same behavior in the same orders of magnitude as for orthogonal orientation. In general, it should be noted that the prototype showed good internal measurement accuracy. The repeated standard deviations were mainly due to the image noise. External influences, such as refraction, had a negligible effect. Furthermore, the method used to detect the object points in the image had a positive influence on the repeatability. On the one hand, the gray values were smoothed by the Canny operator, and on the other hand, multiple image points were used to compute the center of the ellipses, which additionally reduced the impact of noise in the image point measurement.

#### 4.2.2. Absolute Accuracy

The absolute accuracy is indicated by the pose deviation of the stereo camera system with respect to the reference measurement with the laser tracker and is calculated as follows:

(7) $$\sigma =\sqrt{\frac{\sum_{i}^{n}{\left(x_{ref}-x_{act_{i}}\right)}^{2}}{n}}.$$

The reference values result from the reference measurements. For these reference poses, the deviation from the actual values are calculated using the object pose measured by the stereo camera system for each position of the object. The calculation was again performed component-wise. For the orthogonal object alignment, all ten LED markers were included in the evaluation. For the oblique alignments, only eight LED markers were available due to occlusions.

**Figure 15:**
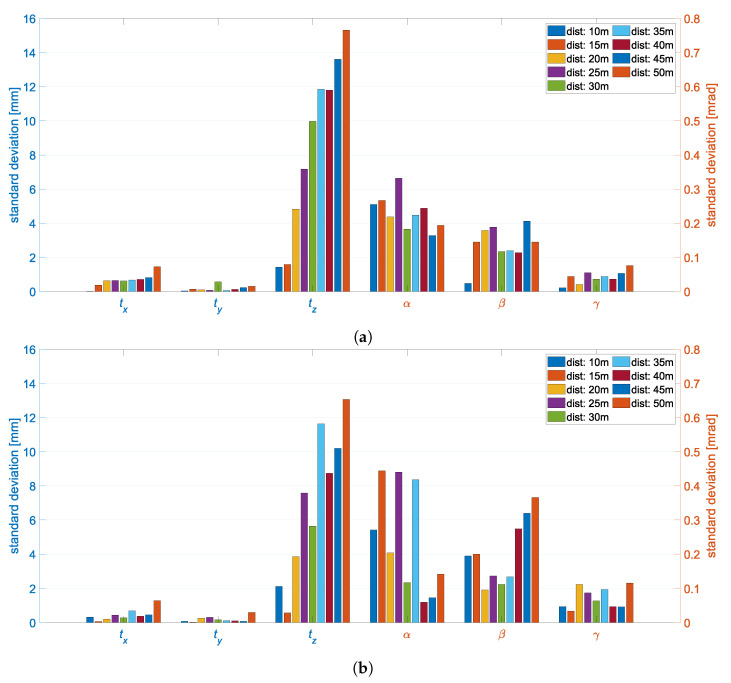
Standard deviations of the object pose from the test measurements at distances varying from 10 m to 50 m. Differentiated by object orientation into orthogonal (**a**) and oblique (**b**) according to the optical axis of the stereo camera system.

Figure 15a visualizes the standard deviations of the object poses of the measurement series with respect to the ground truth values where the object was orthogonally aligned.The translations $t_{x}$ and $t_{y}$ were smaller than 1.5 mm for all distances, whereas the standard deviations in $t_{z}$ reached values of up to approximately 16 mm. This difference resulted from the same effect as in the precision evaluation and was caused by the distance-to-base ratio. Consequently, with larger distances between the object and the stereo camera system, the depth estimation became more uncertain. This resulted in a staircase effect in $t_{z}$. The same staircase effect can also be seen in the translations $t_{x}$ and $t_{y}$, but is less pronounced here due to the measurement of the object points parallel to the image plane. In contrast, the standard deviations of the rotations α, β and γ were in the submillirad range. Hence, the rotations of the object pose were estimated more accurately by the stereo camera system than the translations. The rotation γ shows smaller standard deviations than α and β. This result is in accordance with the Monte Carlo simulation and can be explained by the fact that the depth estimation has no influence on the rotation about the optical axis. The rotation β of the object pose was estimated with higher accuracy than α. A possible explanation for this is the spatial extension of the object markers around the rotation axes. On the measured object, the markers were distributed orthogonally to the *y*-axis by 2 m and orthogonally to the *x*-axis by 1.5 m. The fact that the standard deviations become larger with increasing distance of the object from the stereo camera system can only be determined in γ.

The measurement positions where the object was measured obliquely to the optical axis had similar standard deviations in the object poses (see Figure 15b). For the translation components, the standard deviations were almost identical except for a few outliers. The outliers were due to random effects in the measurement series and not to systematic changes. Due to the rotation of the object about the *y*-axis, the expansion of the LED markers in the image was smaller in width. This affected the standard deviations of the rotations in β and γ, as the rotations are more difficult to estimate due to the smaller distribution of the points in the image. The rotations in β became less accurate especially when the distance was above 40 m. In γ, there was a general increase in the standard deviations over all distances.

The back projection RMSE ranged from 0.4 pixel to 1.0 pixel over all positions. It decreased exponentially with increasing distance. The transformation of the back projection error into object space resulted in an error of 1.1 mm to 2.2 mm. This increased linearly with increasing distance between the object and the stereo camera system. This shows that the back projection error for estimating the object pose is in the order of magnitude of the previously analyzed standard deviations.

Overall, the results of the evaluation are in accordance with the results of the Monte Carlo simulations and show that the high accuracy demands on the system can be met.

## 5. Discussion

We presented a prototype of a generic *stereo camera system* for positioning machines in various environments, e.g., for construction machines or cargo loaders. The conceptual design of the system was based on simulation results, which evaluated different camera configurations and geometric constellations of the measurement object. The results show that the required operating range of 10 m to 50 m can be covered with a single camera system. This resulted in a stereo camera system with a base of 0.5 m, two industrial cameras with 24.5 MP, and two high-resolution lenses with a focal length of 25 mm. The signaling of the object points on the machine is performed by circular LED markers with a diameter of 66 mm. The active markers offer the advantage of better visibility in the sometimes adverse environmental conditions. With the developed software, determination of the machine relative to the stereo camera system is fully automatic without any user interaction. Robust gray value segmentation in the stereo images and a RANSAC-based algorithm for calculating the object pose from the measured image points are used for this purpose.

The marker-based measurement method delivers good results when *estimating the object pose*. The presented results from a test measurement show accuracies of the object pose in the millimeter or submillimeter range. The *x*- and *y*-components of the translation in the camera coordinate system are estimated with especially high accuracy. For distances up to 50 m, the standard deviation is always below 1.5 mm. The standard deviation of the translation in z-direction of the camera coordinate system increases with increasing distance and reaches a value of 16 mm at a distance of 50 m. Hence, along the optical axis, the machine is positioned less accurately, as it is perpendicular to the camera system. With a maximum of 0.4 mrad, the three rotational components of the object pose have no significant influence on the 3D position of a machine. Current positioning systems based on robotic total stations achieve accuracy of a few centimeters. Alone, the 3D point accuracy of real-time object tracking with robotic total stations is a few millimeters under ideal conditions at close range [5]. Depending on the system, however, it can also reach decimeters due to synchronization difficulties [44]. These accuracies can also be achieved on the basis of the test measurements with the stereo camera system in the 1 σ-range.

It was shown that the prototype is able to measure the object pose with a *frequency* of 2 Hz. Image acquisition and transmission via Gigabit Ethernet interface takes about 450 ms. The entire evaluation of a stereo image pair takes about 400 ms on average. This includes the internal data and process management, the detection of the LED markers in the image, and the estimation of the object pose. The prototype thus achieves 2 Hz, but with a latency of 1 s between the measurement of the machine and the output of the corresponding pose of the machine.

The prototype also *robustly determines* the pose of the machine. In a first step, this is ensured within the gray value segmentation of the stereo images. Geometric features of the mapped circles of the LED markers are used to eliminate partially occluded LED markers, which otherwise would falsify the results. Furthermore, apart from the one-time measurement of the LED markers on the machine, no prior information is necessary for estimating the object pose from the measured LED markers. This makes it possible to reliably determine the position of the machine. The use of the RANSAC-based approach also enables a robust estimation of the pose when the proportion of outliers in the data is high.

## 6. Conclusions

In summary, the developed stereo camera system meets the high demands of the industry. The accuracy and the measuring frequency are comparable to those of current systems consisting of robotic total stations, and in some application areas even better. Likewise, it should be pointed out that with the image-based method, only one measurement at a time is required to estimate the object pose. No fusion or synchronization of different sensors is necessary. The cost advantage of the stereo camera system compared to classical systems with robotic total stations is significant.

In future work, it will be interesting to investigate whether the selected focal length can also be used to determine poses of further-away objects, or whether longer focal lengths are required for this purpose. In addition, a method should be developed to reliably determine the exterior orientation of the stereo camera system in a wide variety of applications. In a long-term analysis, the stability of the calibration of the stereo camera system should be investigated. Furthermore, it would be interesting to embed the method into a tracking framework. By this, for example, the threshold values for $C^{\prime }$, $C^{\prime \prime }$, and *F* could be chosen more restrictively because an approximate machine pose would be known from the previous time step. This would improve the speed and the robustness of the pose estimation. Additionally, image acquisition and the segmentation of the markers could be restricted to a reduced image domain, which would further speed up the computations.

## Figures and Tables

**Table 1:** Standard deviations of normal distributed influencing factors in the Monte Carlo simulation.

Variable	Standard Deviation	Source for Derived Magnitudes
3D object point	1 mm	Experience
2D image point	0.1 pixel	[4]
*f*	1.5 μm	[35]
κ	10/m^2^	[17]
$c_{x}$, $c_{y}$	0.01 pixel	[17,36]
$s_{x}$, $s_{y}$	0.1 n m	Experience according to [29]
Base Translation	0.5 m m	[35]
Base Rotation	0.00005 deg	[35]
